# Supplementary material for: Scoping review of knowledge, attitudes, and practices to zoonotic diseases among abattoir workers and residents in proximity to abattoirs in low-middle income countries
Source: PLoS Negl Trop Dis. 2026 Mar 16;20(3):e0013235. doi: 10.1371/journal.pntd.0013235 (PMC13004497; doi:10.1371/journal.pntd.0013235)
Supplement: S1 Appendix — (DOCX) [file pntd.0013235.s001.docx]

**S1_Appendix: List of Abbreviations used In the Manuscript**

1. bTB - Bovine Tuberculosis
2. JBI - Joanna Briggs Institute
3. KAP - Knowledge, Attitudes, and Practices
4. LMICs - Low Middle Income Counties
5. OSF - Open Science Framework
6. PPE - Personal Protective Equipment
7. SOP - Standard Operating Procedure
8. S/N - Serial number
