# Supplementary material for: Scoping review of knowledge, attitudes, and practices to zoonotic diseases among abattoir workers and residents in proximity to abattoirs in low-middle income countries
Source: PLoS Negl Trop Dis. 2026 Mar 16;20(3):e0013235. doi: 10.1371/journal.pntd.0013235 (PMC13004497; doi:10.1371/journal.pntd.0013235)
Supplement: S1 Text — The search strategy presents the search terms and strings used to search PubMed. The strategy was adapted to suit other databases. (DOCX) [file pntd.0013235.s002.docx]

**S1_Text: Search strategy for PubMed**

| Search strategy for PubMed | ("Livestock workers" OR “Farmers” OR "Animal handlers" OR "Animal caretakers" OR “Residents” OR "Community members" OR “Abattoir workers” OR “Butcher*” OR “Veterinarians” OR “meat inspectors” OR “animal handlers” OR “slaughterhouse workers” OR “meat processing workers” OR “meat handlers” OR “Occupational exposure”)  AND  ("Zoonotic diseases" OR Zoonoses OR “Zoonosis” OR "Infectious diseases" OR "Disease transmission" OR "Disease prevention"  OR Brucellosis* OR erysipel* OR Listeri* OR anthra* OR leptospi* OR staphylo* OR tetan* OR tubercul* OR mycobacterium OR clamyd* OR coxiel* OR Q-fever OR dermatoph* OR louping* OR pseudocowpox OR cowpox OR “foot and mouth disease” OR FMD OR scabies OR aspergil* OR “hemorrhagic virus” OR “haemorrhagic virus” OR “tick borne virus” OR “tick-borne virus” OR tularem* OR “animal borne disease*” OR “animal-borne disease*”)  AND  ("Knowledge and practices" OR “Knowledge” OR “Awareness” OR “Understanding” OR “Practices” OR “Behavior” OR “Behaviour” OR “Attitudes” OR “Beliefs” OR “health practices” OR “health knowledge” OR “Act”)  AND  ("Afghanistan" OR "Benin" OR "Burkina Faso" OR "Burundi" OR "Central African Republic" OR "Chad" OR "Comoros" OR "Democratic Republic of the Congo" OR "Eritrea" OR "Ethiopia" OR "Gambia" OR "Guinea" OR "Guinea-Bissau" OR "Haiti" OR "Democratic People's Republic of Korea" OR "Liberia" OR "Madagascar" OR "Malawi" OR "Mali" OR "Mozambique" OR "Niger" OR "Rwanda" OR "Sierra Leone" OR "Solomon Islands" OR "Somalia" OR "South Sudan" OR "Sudan" OR "Syria" OR "Tajikistan" OR "Togo" OR "Tuvalu" OR "Uganda" OR "United Republic of Tanzania" OR "Yemen" OR "Zambia" OR "Angola" OR "Bangladesh" OR "Bhutan" OR "Bolivia" OR "Cabo Verde" OR "Cambodia" OR "Cameroon" OR "Chad" OR "Cote d'Ivoire" OR "Djibouti" OR "Egypt" OR "El Salvador" OR "Eswatini" OR "Fiji" OR "Ghana" OR "Guatemala" OR "Honduras" OR "India" OR "Indonesia" OR "Kenya" OR "Kiribati" OR "Kyrgyz Republic" OR "Lao People's Democratic Republic" OR "Lesotho" OR "Mauritania" OR "Micronesia" OR "Moldova" OR "Mongolia" OR "Morocco" OR "Myanmar" OR "Nepal" OR "Nicaragua" OR "Nigeria" OR "Pakistan" OR "Papua New Guinea" OR "Paraguay" OR "Philippines" OR "Samoa" OR "Senegal" OR "Solomon Islands" OR "South Africa" OR "Sri Lanka" OR "Sudan" OR "Syria" OR "Tajikistan" OR "Tanzania" OR "Timor-Leste" OR "Tonga" OR "Tunisia" OR "Turkmenistan" OR "Ukraine" OR "Uzbekistan" OR "Vanuatu" OR "Vietnam" OR "West Bank and Gaza" OR "Zambia") AND ("Developing Countries" OR "Income" OR "Low-Income Countries" OR "Lower Middle-Income Countries") |
| --- | --- |
