# Supplementary material for: Scoping review of knowledge, attitudes, and practices to zoonotic diseases among abattoir workers and residents in proximity to abattoirs in low-middle income countries
Source: PLoS Negl Trop Dis. 2026 Mar 16;20(3):e0013235. doi: 10.1371/journal.pntd.0013235 (PMC13004497; doi:10.1371/journal.pntd.0013235)
Supplement: S1 Table — The table of study characteristics presents the information extracted from the 16 included articles. (DOCX) [file pntd.0013235.s003.docx]

**S1_Table: Study characteristics**

| **Author/year** | **Journal** | **Study design** | **Sample Size** | **Country** | **Type of Zoonosis** | **Knowledge** | **Attitudes** | **Practices** |
| --- | --- | --- | --- | --- | --- | --- | --- | --- |
| Brown, 2011 | Occupational and Environmental medicine | Cross-sectional | 110  (M=74, F=36) | Jamaica | Leptospirosis | Ninety-one (83%) respondents correctly noted that the disease can be transmitted from animals to man, while 78% of the study group had heard about leptospirosis.  Of this proportion, 43 indicated that they learnt about the disease from a public health inspector and 37 learnt it through the print and mass media. | The majority (59%) of studied workers believed that leptospirosis could be cured. Personal protective gear (gowns/ aprons, boots, gloves and hats) and use of clean gear at the start of each day were thought to be very important by more than 80% of respondents. In addition, almost all respondents indicated that hand washing was an important safety procedure after soiling with body fluids from animals in the work environment. | Most (90%) of workers had health certification |
| Rosiji, 2012 | Global Veterinaria | Cross-sectional | 437  (M =315, F=122) | Nigeria | Bovine tuberculosis | Not indicated | Not indicated  More people in the age group 30-39 years and the males exhibited good measures towards bovine TB prevention | 437 (96.6%) butchers said that they allowed veterinarians to carry out routine meat inspection on their animals. The practices of good hygiene, hand washing and wearing of protective materials such as gloves and boots were observed. On limiting cattle to cattle spread of bovine tuberculosis, the butchers said they employed early isolation of infected animals for treatment, avoidance of close contact with the infected animals.  76 (36.7%) respondents in the age group 30-39 years and the males exhibited good measures towards |
| Hambolu*,* 2013 | Plos One | Cross-sectional | 349  (M=273, F=76) | Nigeria | Bovine tuberculosis | 294 (84.2%) respondents had heard of bTB but only 31% knew that it could spread from animals to humans (bTB) and less than 20% were aware that even healthy looking meat could be contaminated | Not indicated | 75 (21.5%) participants consumed fuku elegushi (meat) which predisposes them to TB. 310 (88.8%) participants did not wear gloves while processing meat, ate raw meat, sold meat even when visibly contaminated and did not wash hands after processing raw meat. |
| Tsegay*,* 2017 | BMC Infectious Diseases | Cross-sectional | 210  (M= 117, F =93) | Ethiopia | Nonspecific | Not indicated | Not indicated | Majority claimed using gloves and covering their mouth during slaughtering and eviscerating process. Majority of the respondents consumed raw dairy products and meat, and accidentally cut themselves during slaughtering and eviscerating. |
| Cook*,* 2017 | BioMed Central | Cross-sectional | 738  (M=716, F=22) | Kenya | Nonspecific | Thirty-one percent of the 738 slaughterhouse workers knew that disease could be transmitted from animals. Forty-two percent knew that meat could be a source of disease. Only 8% of workers could name a zoonotic disease. | Not indicated | Fifty-three percent of workers reported wearing protective clothing |
| Mostafavi*,* 2017 | International Journal of Infectious Diseases | Cross-sectional | 190  (M=190, F=0) | Iran | Crimean-Congo haemorrhagic fever disease | Not indicated | Not indicated | 143 (75.3%) workers had a history of being splashed with fluids of animals viscera for more than once on their faces and 152 (80%) on other parts of their bodies. 49 (25.8%) butchers had a history of cutting their hands or other parts of their bodies at least once during their work and 17.4% recalled an ectoparasite bite during the last year. Majority of the participants had never applied chemical disinfectant to disinfect their knives, hands and faces. Some participants did not use any personal protective equipment (mask, gloves, overalls or boots), |
| Awah-Ndukum, 2018 | BMC infectious diseases | Cross sectional | 107  (M= 96, F= 11) | Cameroon | Brucellosis | Not indicated | Not indicated | 59 (55%) respondents had regular contact with domestic ruminants (cattle, sheep and goats), regularly consumed unpasteurised milk, assisted in dressing of slaughtered animals and manipulated aborted foetuses and other uterine contents without using personal protective equipment such as gloves. Despite suffering previous miscarriages, Brucella IgG seropositive pregnant women in the study were still involved in the abattoir activities.  All Brucella IgG seropositive respondents did not use of personal protective equipment such as gloves during work. |
| Ekere*,* 2018 | Veterinary World | Cross-sectional | 137  (M=103, F=34) | Nigeria | Brucellosis | 93 (67.9%) slaughterhouse workers had not heard of brucellosis. | Not indicated | The workers (97; 70.8%) were massively involved in practices that could aggravate spread of Brucella infection such as non-use of PPE during slaughterhouse operations, disposal of slaughterhouse wastes, including eviscerated foetuses and pregnant uterine contents, by open-air dump method (64.9%) and illegal sale of eviscerated foetuses for human consumption (59.9%) or preparation of dog food (71.5%). |
| Fekadu*,* 2018 | Frontiers in Veterinary Science | Cross-sectional | 300  (M=241, F= 59) | Ethiopia | Bovine tuberculosis | 286 (95.3%) respondents knew about TB transmission from animals to humans 95% heard about bTB and 93% knew that eating raw meat could be a source of bTB for humans. More than 62.7% of the respondents in the high risk group strongly agreed that contracting bTB would prevent them from coming to work, keep them in bed for an extended period of time and cause death. | Not indicated | Most participants did not wear PPE during work hours and 292 (97.3%) were found to consume raw meat. |
| Madut*,* 2019 (a) | Frontiers in public health | Cross-sectional | 598  (M= 326, F = 272) | South Sudan | Brucellosis | Most participants recognised the disease once clinical signs were extensively explained. Once the disease was fully discussed, they got the local names for this condition. This highlighted the fact that they knew the disease, but possibly not the risk it posed to them. Female respondents, regardless of risk group were more aware of the zoonotic diseases compared to their male counterparts. | The views from focus group discussion with farmers revealed that they believed food sellers, butchers and herders were in more contact with raw meat than anybody else in the community | There are basic norms taught to every generation in most of these communities, the norms and practices inadvertently protect the people against infections including zoonotic diseases. Women were excluded from information dissemination activities such as; local meetings, health intervention and planning |
| Madut*,* 2019 (b) | BMC infectious diseases | Cross-sectional | 234  (M= 209, F= 25) | South Sudan | Brucellosis | 83 (35.5%) respondents knew about zoonotic diseases | Not indicated | 74 (31.9%) used personal protective gears at work. 189 (84.6%) consumed raw animal products. 157 (67.1%) washed hands after work. |
| Odetokun*,* 2020 | Food Protection Trends | Cross-sectional | 203  (M= 112, F= 91) | Nigeria | Nonspecific | 129 (72.5%) knew that slaughterhouse workers can get diseases from animals. 151 (84.8%) knew that diseases can come from the slaughterhouse environment and from meat (135; 75.8%). | Although, the majority of the respondents (> 70%) were aware that slaughterhouse workers can get diseases from animals, from the slaughterhouse environment and from meat, the effect of this awareness was not seen in the practice of hygiene and work-related ergonomics | 58 (32.6%) respondents reported regular usage of PPE, with workers demonstrating poor personal hygiene. |
| Agbalaya*,* 2020 | Veterinary World | Cross-sectional | 156  (M= 121, F= 35) | Nigeria | Bovine tuberculosis | Respondents who did not know the mode of bTB transmission were 2.2 times more at risk of exposure than those with requisite knowledge | Not indicated | Respondents who had spent more than 6 years in livestock handling were associated with 3.1 times increased risk of exposure to bTB infection.  Sleeping in animal shed was associated with increased risk of bTB compared to those that did not. |
| Bahiru*,* 2022 | Ethiopian Journal of Health Development | Cross-sectional | 113  (M= 91, F= 22) | Ethiopia | Zoonotic tuberculosis | 73 (64.6%) respondents did not know the main hygiene practices in the abattoir. | Not indicated | 105 (92.9%) respondents had the habit of consuming raw milk/meat and had contact with live animals and animal products for more than eight hour’s daily  None of the abattoir workers spontaneously responded to the use of sanitisers as one of the hygiene practices. |
| Njoga*,* 2023 | PLOS ONE | Cross-sectional | 157  (M = 157, F =0) | Nigeria | Bovine tuberculosis  Contageous bovine pleuro-pneumonia  Fascioliasis (by *Fasciola hepatica*)  Cysticercosis  Liver abscess | 81 (51.6%) respondents knew some food-producing animals can harbour meat-borne pathogens;  84 (53.5%) knew that non-use of PPE can enhance the transmission of zoonotic pathogens among slaughterhouse workers.  97 (61.8%) knew that human infections with zoonotic meat-borne pathogens could result from the use of contaminated water for carcass/meat processing during slaughterhouse operations  119 (75.8%) knew that eating or drinking while processing carcass especially with unwashed hands may increase the chance of infections with zoonotic pathogen | Not indicated | Practices included: Using the same water to wash more than one carcass (82; 52.2%), using PPE (44; 28%) , dressed carcass on bare slaughterhouse floor (112; 71.3%). |
| Sint, 2023 | PLOS ONE | Cross-sectional | 139  (M= 119, F = 20) | Myanmar | *Toxoplasmosis godii* infection | 75 (54%) respondents had high knowledge level regarding *T.gondii* infection | Not indicated | 27 (19.4%) of 139 respondents ate raw meat.  More than half 59.3% of slaughterhouse workers did not wear PPE when slaughtering animals 123 (88.5%) came in contact with animal organs, muscles or blood. 101(85.6%) wash hand before and after eating. |
