# Supplementary material for: Scoping review of knowledge, attitudes, and practices to zoonotic diseases among abattoir workers and residents in proximity to abattoirs in low-middle income countries
Source: PLoS Negl Trop Dis. 2026 Mar 16;20(3):e0013235. doi: 10.1371/journal.pntd.0013235 (PMC13004497; doi:10.1371/journal.pntd.0013235)
Supplement: S2 Table — It captures the reason for the exclusion of six studies. (DOCX) [file pntd.0013235.s004.docx]

**S2_Table: Included and excluded articles with reasons for exclusion**

| **Serial Number** | **Title** | **Remark** | **Reason for Exclusion** |
| --- | --- | --- | --- |
|  | Brown PD, McKenzie M, McGrowder D. Environmental risk factors associated with leptospirosis among butchers and their associates in Jamaica. Int J Occup Environ Med. 2011; 2(1):47-57. (Ref 19) | Included | Not applicable |
|  | Rosiji CO, Adesokan, H.K. (2012). Measures taken for bovine tuberculosis prevention among butchers in two major abattoirs in Ibadan, South-western Nigeria. Global Veterinari 2012; 9(1): 107-112 - DOI: 10.5829/idosi.gv.2012.9.1.64139 (Ref 20) | Included | Not applicable |
|  | Hambolu D, Freeman J, Taddese HB. Predictors of bovine TB risk behaviour amongst meat handlers in Nigeria: a cross-sectional study guided by the health belief model. PloS One 2013; 8(2): e56091 DOI: 10.1371/journal.pone.0056091. (Ref. 21) | Included | Not applicable |
|  | Cook EAJ, De Glanville WA, Thomas LF, Kariuki S, Bronsvoort BMDC. Fèvre EM. Working conditions and public health risks in slaughterhouses in western Kenya. BMC Public Health 2017; 17(1):1-12- doi: 10.1186/s12889-016-3923-y. (Ref 22) | Included | Not applicable |
|  | Mostafavi E, Pourhossein B, Esmaeili S, Bagheri AF, Khakifirouz S, Shah-Hosseini N, et al. Seroepidemiology and risk factors of Crimean-Congo Hemorrhagic Fever among butchers and slaughterhouse workers in southeastern Iran. Int J Infect Dis. 2017; 64:85-89. doi: 10.1016/j.ijid.2017.09.008. Epub 2017. (Ref 23) | Included | Not applicable |
|  | Tsegaye D, Gutema FD, Terefe Y. Zoonotic diseases risk perceptions and protective behaviors of consumers associated with consumption of meat and milk in and around Bishoftu, Ethiopia. Heliyon 2022; 8(8):. DOI: 10.1016/j.heliyon. 2022.e10351 (Ref 24) | Included | Not applicable |
|  | Awah-Ndukum J, Mouiche MMM, Kouonmo-Ngnoyum L, Bayang HN, Manchang TK, Poueme RSN, et al. Seroprevalence and risk factors of brucellosis among slaughtered indigenous cattle, abattoir personnel and pregnant women in Ngaoundéré, Cameroon. BMC Infectious Diseases 2018; 18(1). doi: 10.1186/s12879-018-3522-x (Ref 25) | Included | Not applicable |
|  | Ekere SO, Njoga EO, Onunkwo JI, Njoga UJ. Serosurveillance of Brucella antibody in food animals and role of slaughterhouse workers in spread of Brucella infection in Southeast Nigeria. Veterinary World 2018; 11(8): 1171-1178. doi: 10.14202/vetworld.2018.1171-1178. (Ref 26) | Included | Not applicable |
|  | Fekadu F, Beyene TJ, Beyi AF, Edao BM, Tufa TB. Woldemariyam FT; et al. Risk Perceptions and Protective Behaviors Toward Bovine Tuberculosis Among Abattoir and Butcher Workers in Ethiopia. Frontiers in veterinary science 2018; 5(0):169. doi: 10.3389/fvets.2018.00169. (Ref 27) | Included | Not applicable |
|  | Madut NA. Muleme J. Kankya C. Nasinyama GW. Muma JB, Godfroid J, et al. (2019a). The epidemiology of zoonotic brucellosis in Bahr el Ghazal region of South Sudan. Frontiers in Public Health 2019a; 7; DOI: 10.3389/fpubh.2019.00156 (Ref 28) | Included | Not applicable |
|  | Madut NA, Ocan M, Muwonge A, Muma JB, Nasinyama GW, Godfroid J, et al. Sero-prevalence of brucellosis among slaughterhouse workers in Bahr el Ghazal region, South Sudan. BMC infectious diseases 2019b; 19(1): DOI: 10.1186/s12879-019-4066-4 (Ref 29) | Included | Not applicable |
|  | Agbalaya MA, Ishola OO, Adesokan HK, Fawole OI. Prevalence of bovine tuberculosis in slaughtered cattle and factors associated with risk of disease transmission among cattle handlers at Oko-Oba Abattoir, Lagos, Nigeria. Veterinary World 2020; 13(8): 1725-1731 doi: 10.14202/vetworld.2020.1725-1731. (Ref 30) | Included | Not applicable |
|  | Odetokun IA, Ghali-Mohammed I, Alhaji NB, Nuhu AA, Oyedele HA, Ameen SA. Occupational health and food safety risks in Ilorin, Northcentral Nigeria: A cross-sectional survey of slaughterhouse workers. Food Protection Trends 2020; 40(4) 241-250. (Ref 31) | Included | Not applicable |
|  | Bahiru G, Jena PK, Addissie A, Behera MR, Fromsa A, Gumi B. Zoonotic Tuberculosis in Occupationally Exposed Groups in the Adama Municipal Abattoir, Central Ethiopia. Ethiopian Journal of Health Development 2022; 36(1): doi: 10.20372/ejhd.v36i1.5001. (Ref 32) | Included | Not applicable |
|  | Njoga EO, Ilo SU, Nwobi OC, Onwumere-Idolor OS, Ajibo FE, Okoli CE. (2023). Pre-slaughter, slaughter and post-slaughter practices of slaughterhouse workers in Southeast, Nigeria: Animal welfare, meat quality, food safety and public health implications. PLoS One 2023; 18(3): DOI: 10.1371/journal.pone.0282418 (Ref 33) | Included | Not applicable |
|  | Sint NH, Htun YM, Win TT, Mon AS, Lwin TZ, Maung LO, et al.. Seroprevalence and associated risk factors of Toxoplasma gondii infection among slaughterhouse workers in Yangon Region, Myanmar: A cross-sectional study. PLoS ONE 2023; 8(4): e0284352. DOI: 10.1371/journal.pone.0284352. (Ref 34) | Included | Not applicable |
|  | Zulu VC, Syakalima M, Ali J. Ethical dimensions of zoonotic disease research: Perspectives of traditional livestock keepers in Zambia. Wellcome Open Research 2023, 7, 201. | Excluded | The study outcome, was different from our eligibility criteria |
|  | Durrance-Bagale A, Rudge JW, Singh NB, Belmain SR, Howard N. Drivers of zoonotic disease risk in the Indian subcontinent: A scoping review. One Health 2021, 13, 100310. | Excluded | This study is a scoping review. We only wanted primary studies. |
|  | Nenzhelele F, Anyanwu FC, Ramoteme M, Mabunda J, Henry A, Kwabena K, A quantitative assessment of the level of knowledge, attitude and practices of farm workers regarding schistosomiasis in a rural community in South Africa. African Journal of Primary Health Care and Family Medicine 2020, 12(1), 1-8. | Excluded | The study’s focus was on schistosomiasis but our study was on livestock. |
|  | Musallam II, Abo-Shehada MN, Hegazy YM, Holt HR, Guitian FJ. Systematic review of brucellosis in the Middle East: disease frequency in ruminants and humans and risk factors for human infection. Epidemiology & Infection 2016, 144(4), 671-685. | Excluded | This study is a systematic review. We needed primary studies |
|  | Adesokan, HK., Ocheja SE. Knowledge, attitudes and practices of traceability among livestock traders in south-western Nigeria: implications for sustainable livestock industry. Tropical animal health and production 2014, 46(1), 159-165. | Excluded | The study outcome, was different from our eligibility criteria. It focused on traceability and the practice of providing feedback on diseases encountered in slaughtered animals from the abattoir to the farm. |
|  | Aworh MK, Okolocha E, Kwaga J, Fasina F, Lazarus D, Suleman I, et al. Human brucellosis: seroprevalence and associated exposure factors among abattoir workers in Abuja, Nigeria-2011. The Pan African Medical Journal 2013, 16, 103. | Excluded | The study outcome was different from our eligibility criteria. The study focused on exposure and seroprevalence of Brucellosis. |
