# Supplementary material for: Scoping review of knowledge, attitudes, and practices to zoonotic diseases among abattoir workers and residents in proximity to abattoirs in low-middle income countries
Source: PLoS Negl Trop Dis. 2026 Mar 16;20(3):e0013235. doi: 10.1371/journal.pntd.0013235 (PMC13004497; doi:10.1371/journal.pntd.0013235)
Supplement: Attachment — Submitted filename: RESPONSES TO REVIEWERS.docx [file pntd.0013235.s007.docx]

**RESPONSES TO REVIEWERS’ COMMENTS**

We are very grateful for this crucial comments made by the reviewers to bring the best out of our work. We have corrected and responded to the comments one-by-one in the table for clarity and easy location in the manuscript.

| **S/N** | **Reviewer** | **Reveiwer’s comment** | **Authors’ Response** |
| --- | --- | --- | --- |
| **METHODS** | | | |
| **S/N** | **Reviewer** | **Comment** | **Response** |
| 1. | **Reviewer #1** | The authors fail to define ‘residents near slaughterhouses’ operationally, leaving the notion of ‘proximity’ unclear—whether it refers to geographic distance, duration of exposure, or functional interaction. | We agree that the operational definition of proximity is essential for clarity. Proximity referred to is geographic distance. We used terms like ‘residents living near abattoirs’ and nearby residents. These terms are indicative of geographic locations rather than duration of exposure or functional interaction. We have clearly defined this term in the revised Methods section, subsection search strategy (Lines 125-128) |
| 2. |  | While the authors indicate adherence to JBI guidelines for scoping reviews, which do not mandate formal quality appraisal, omitting any assessment of study quality restricts the interpretability of the findings. | The study was conducted as a scoping review and we followed the Joanna Briggs Institute (JBI) whose methodology does not mandate formal quality appraisal of included studies because the primary aim of a scoping review is to map available evidence, rather than to evaluate study quality. However, we accept that without a formal appraisal the interpretability of the findings may be limited. To address this, we have revised the manuscript to acknowledge the concern as a limitation in the discussion section (Lines 383-385).  We have also clearly indicated in the methodology section (subsection critical appraisal of included studies) that no critical appraisal of the included studies was conducted (Line 156-158) |
| 3. | **Reviewer #3** | The methodology of the papers selections is not clear. Which were the key words used to select the papers? | We appreciate you pointing this out. To improve clarity of the manuscript, we have revised the methods section to state the exact keywords and the search strings adopted in each database. We have also stated and provided the Supplementary Information (SI_1) (Line 121-125). |
| 4. |  | There´s no statistics once it´s a descriptive study. | Yes. That’s correct. As a scoping review, our study primarily employed descriptive and narrative synthesis, consistent with the JBI guide. We have clarified this in the revised methods section (lines 151-151). |
| 5. |  | Ethical approval was referred. | Considering our study is a scoping review of published literature, no primary data were collected from human participants, therefore, ethical approval was not required. To improve clarity we have revised the manuscript by adding a subsection that clearly states that ethical approval was not applicable. (Lines 162-163) |
| **RESULTS AND DISCUSSION** | | | |
| **S/N** | **Reviewer** | **Comment** | **Response** |
| 6. | Reviewer 1 | In the knowledge-related findings (lines 226–239) (initial manuscript line numbers), the authors report percentages without clearly linking them to the corresponding studies. | References have been made to the indicated percentages. See lines 240 to 241. Other lines where percentages were indicated already have references. |
| 7. |  | The section on practices (lines 252–273) (initial manuscript line numbers), presents issues as well, combining data on PPE use, raw product consumption, and hygiene without differentiating the relevance of each practice across various zoonotic diseases. | The section has been edited and aligned accordingly. See lines 264-281 |
| 8. |  | Although the discussion is broad in scope, some interpretations exceed the evidence presented. For example, the claim of a “disconnect between knowledge and behaviour” (lines 287–288) (initial manuscript line numbers), lacks support from statistical analyses, as most studies evaluated either knowledge or behaviour but not both simultaneously. | One of the included studies considered knowledge, attitude and practice, and provided statistical evidence (Odetokun *et al.,* 2020). The study reported disconnection between knowledge and practice. The study observed that slaughterhouse workers knew they could get diseases from animals (129; 72.5%), from the slaughterhouse environment (151;84.8%) and meat (135;75.8%) but only 32.6% respondents reported regular usage of PPE. Also, Madut *et al.,* 2019b reported 35.5% had knowledge about zoonotic diseases but 84.6% participants consumed raw animal products. See lines 294-296 |
| 9. |  | Similarly, the discussion on cultural factors and social norms (lines 302–305) (initial manuscript line numbers), relies heavily on the interpretations of primary study authors, without critically examining how these factors were measured or whether such interpretations are consistent across the nine culturally diverse countries included in the review. | The sentences have been recast to reflect that the statements are more of personal understanding/observations during the primary studies. See line 311 |
| **CONCLUSION** | | | |
| **S/N** | **Reviewer** | **Comment** | **Response** |
| 10. | Reviewer #1 | Figure 3 (line 317), which presents the conceptual framework, provides a useful visual tool but has important limitations. The proposed causal relationships lack empirical support from the included studies, many of which did not test such associations. In addition, the framework does not adequately integrate structural or policy-level determinants, despite their identification in the discussion as key influences on worker practices. | Figure 3 has been reconstructed to capture the empirical findings and the references..  The studies tested for associations which was the reason they were included causal relationships by the authors.  lines 345-368 |
| 11. |  | The synthesis section needs a more critical reflection on the limitations of the primary studies. For example, the overrepresentation of studies from Nigeria and Ethiopia raises concerns about the generalizability of the findings to other low- and middle-income settings. Moreover, while the absence of studies on residents near slaughterhouses is noted, the authors do not fully explore the implications of this gap for public health policy. | The seeming over-representation of studies from Nigeria and Ethiopia shows that more studies have been done in these countries than others. It also highlights the need for studies on this subject area to be conducted in more LMICs. |
| 12. | Reviewer #2 | Recommendations need to be more specified and showing examples of the intervention and the address the urgent behaviors that need to be improved. | This has been addressed. See lines 388 and 390 |
| 13. | Reviewer #3 | The conclusions are easily discussed once they is already expected for anyone who works in the field. It´s not possible to evaluate the conclusions without the keywords used. Maybe the number of studies could be increased if new keywords are included. The public health relevance is addressed. | Comment acknowledged. |
| EDITORIAL AND DATA PRESENTATION MODIFICATION | | | |
| **S/N** | **Reviewer** | **Comment** | **Response** |
| 14. | Reviewer #2 | Regarding the title:" and residents in proximity to abattoirs" it is better to be removed as there is no publications assessed KAP among this group. | We started off with the topic and we searched for studies on residents in proximity to abattoirs. Removing it from the title at this point will affect our search strategy and other sections of our study. |
| 15. |  | Regarding the introduction: it needs more declaration of the effect of zoonotic diseases on public health. | More declarations have been added. See lines 73-75, and 88-89 |
| 16. |  | Methods: The link shown in line 101 is not correct it needs to be modified. | The link has been corrected. It now opens to the protocol. See line 106. |
| 17. |  | line 115: the publication are from 2010 not 2000. | This has been corrected. See Line 119. |
| 18. |  | line 124: comma after if they were should be removed. | The comma has been removed. |
| 19. |  | line 202: sample size should be from 107 not 110. | It has been corrected to 107. See line 214 |
| 20. |  | line 253: what does SH refer to? | Corrected to slaughterhouse |
| 21. |  | Figure 2 why 2024 is included while the studies are till 2023 and it should be titled number of publications from 2010 to 2023. | 2024 has been removed. The width now covers two years and the figure represented. See line 212. |
| 22. |  | Table 2 is too long it is better to be divided into two tables. | The authors think that it should be left as one table because breaking it into two tables would cover one and a half pages each and would not make a difference. |
| 23. |  | In the discussion section it needs to be mentioned why attitude is assessed in only 3 papers. | This has been include in Lines 290-291. The other authors did not set out to assess attitude in their studies. |
| 24. | Reviewer #3 | The inclusion of key words is crucial for the study to accurately evaluate its conclusions. The authors would consider including other keywords to increase the number of studies. | Two more keywords have been added: ‘LMICs’ and ‘scoping review’ (see lines 57 and 58) |
| **SUMMARY AND GENERAL COMMENTS** | | | |
| 25. | Reviewer #1 | To enhance the manuscript, the authors should expand the methodology to clarify how study limitations informed the synthesis; reorganize the results to enable clearer cross-study comparisons; revise the conceptual framework to align more closely with the available evidence; and deepen the discussion of research and policy implications, especially regarding underserved populations in low- and middle-income countries. | This has been done and it spans through the manuscript |
| 26. |  | It is better to add list of abbreviations and avoid mentioning the abbreviation without the full name in the first time. | All abbreviations have their full name preceding them. Furthermore, a list of the abbreviations used in the manuscript has been produced and added to the supplementary information |
