# Supplementary material for: Scoping review of knowledge, attitudes, and practices to zoonotic diseases among abattoir workers and residents in proximity to abattoirs in low-middle income countries
Source: PLoS Negl Trop Dis. 2026 Mar 16;20(3):e0013235. doi: 10.1371/journal.pntd.0013235 (PMC13004497; doi:10.1371/journal.pntd.0013235)
Supplement: Attachment — Submitted filename: RESPONSES TO REVIEWERS_2.docx [file pntd.0013235.s008.docx]

**RESPONSES TO REVIEWERS’ COMMENTS**

We are very grateful for more comments made by the reviewers to bring the best out of our work. We have corrected and responded to the comments one-by-one in the table for clarity and easy location in the manuscript.

| **s/n** | **Section** | **Reviewer’s Comment** | **Authors’ Respnse** |
| --- | --- | --- | --- |
| 1. | Discussion | Reviewer #2: in discussion section expand on why attitudes were the least studied domain? | This has been expanded. See lines 291-298 |
