# Supplementary material for: Scoping review of knowledge, attitudes, and practices to zoonotic diseases among abattoir workers and residents in proximity to abattoirs in low-middle income countries
Source: PLoS Negl Trop Dis. 2026 Mar 16;20(3):e0013235. doi: 10.1371/journal.pntd.0013235 (PMC13004497; doi:10.1371/journal.pntd.0013235)
Supplement: Attachment — Submitted filename: Response to Reviewers docx.docx [file pntd.0013235.s009.docx]

**RESPONSE TO REVIEWERS**

Thank you for the keen observations. We are very grateful for all comments made to bring the best out of our work. We have corrected and responded to the comments one-by-one in the table for clarity and easy location in the manuscript.

| **Editorial’s Comments** | **Responses** |
| --- | --- |
| Please check the use of singular vs plural for ‘attitudes’ and ‘practices’ – typically both should be in plural throughout the text. | Attitude has been changed to attitudes (lines 1, 58, 63, 228, 254, Table 2/ first row/ column 5, 291, within the conceptual framework, 553, 555, 557)  However, the use of ‘attitude’ in some instances are correct. For example, the statement “Attitude assessments typically require longer questionnaires, Likert scale items, or qualitative methods, increasing costs and field time…” It would be grammatically incorrect to use “Attitudes assessments….”.  Practice has been changed to practices (lines 51, 58, 63, 228, 265, Table 2/ first row/ column 4, 306, within the conceptual framework, 553, 555, 557). |
| In Table 1, the first column is indicated to tell Name of first author and year of publication, but it includes ‘et al.’ as well as name of all authors when there was two authors. | The correction has been done in Tables 1 and 2. |
| Use of capital first letters needs further check. Why currently capital letter for Pleuropneumonia, why not for cysticercosis?  Crimean-Congo hemorrhagic fever is written with capital letters for both ‘Crimean’ and ‘Congo’, and with hyphen, by e.g. WHO (Table 1, Table 2). | The corrections have been made.  Table 1/row 7/column 4, Table 2/row 7 /column 3, lines 222, 380,  ‘Crimean-Congo haemorrhagic fever disease’ (this is the standard way of writing this disease because it was first isolated in these two locations and the disease was named after them).  ‘Cysticercosis’ (Table 1/row 16/column 4, Table 2/row 16/column 3  ‘Bovine pleuropneumonia’ (Table 1/last row /column 4, Table 2/row 16/column 3 |
| Toxoplasma gondii needs to be written with capital first letter for genus name, and Italics for both genus and species name, in both text and Table 1. | This has been corrected:  Table 1/last row/column 4, Table 2/16/columns 3 and 4. lines 223, 527. |
| In Table 2, please check and clarify, these two sentences leave confusion: “292 (97.3%) participants of 300 were found to consume raw meat. 225 (75%) had eaten or consumed raw meat” | The point has been restated (Table 2/9/ column 6) |
| Consider and check the use of abbreviations.  Are e.g. SH-worker and bTB needed?    Currently bTB is explained two times. | SH has been written as slaughterhouse throughout the document (Table 2: 8/column 6, 5/columns 4 and 6; and 16/column 6)  We used bTB as abbreviation for bovine tuberculosis. We used the abbreviation (it is a standard abbreviation) because we referred to it severally in the manuscript.  We could not locate where we explained bTB two times. |
| In list of included studies, word ’applicable’ is misspelled.  Is S/N 4. included? There is no remark for that one.  Abbreviation “S/N” is not explained in the document. | The spelling has been corrected.  S/N 4 is included and it has been indicated.  S/N has been added to the abbreviation list. (line 572) |
| American and British spelling are currently mixed (e.g. minimize; behaviour), please check for consistent style. | Thank you so much for this observation. All spellings have been changed to British.  Analyse (lines 63, 156)  Minimise (line 129)  Metres (line 128)  Summarised (line 152)  Synthesised (line 152)  Recognised (243, 249, 252, 261, table 2/1/column 4, table 2/10/column 4, 260)  Unpasteurised (line 272, Table 2/6/column 6, 320)  Foetuses (line 278, Table 2/7/column 6, Table 2/8/column 6)  Emphasised (Table 2/1/column 5)  Practise (as a verb) Table 2/3/column 6  Sanitisers (Table 2/14/column 6)  Programmes (316, 341, 415,  Behaviour is British spelling |
